# High prevalence of abnormal menstruation among women living with HIV in Canada

**DOI:** 10.1371/journal.pone.0226992

**Published:** 2019-12-27

**Authors:** Christina Valiaveettil, Mona Loutfy, V. Logan Kennedy, Sheila Caddy, Mark Yudin, Tracey Conway, Erin Ding, Paul Sereda, Alexandra de Pokomandy, Angela Kaida, for the CHIWOS Research Team

**Affiliations:** 1 Schulich School of Medicine and Dentistry, Western University, London, ON, Canada; 2 Faculty of Medicine, University of Toronto, Toronto, ON, Canada; 3 Women’s College Research Institute, Women’s College Hospital, Toronto, ON, Canada; 4 Department of Obstetrics and Gynecology, University of Alberta, Edmonton, AB, Canada; 5 Department of Obstetrics and Gynecology, St. Michael’s Hospital, Toronto, ON, Canada; 6 British Columbia Centre for Excellence in HIV/AIDS, Vancouver, BC, Canada; 7 Chronic Viral Illness Service, McGill University Health Centre, Montreal, QC, Canada; 8 Department of Family Medicine, McGill University, Montreal, QC, Canada; 9 Faculty of Health Sciences, Simon Fraser University, Burnaby, BC, Canada; University of Alabama at Birmingham, UNITED STATES

## Abstract

**Objectives:**

To measure the prevalence and correlates of abnormal menstruation among women living with HIV (WLWH) in Canada.

**Methods:**

We used cross-sectional questionnaire data from the community-based Canadian HIV Women’s Sexual and Reproductive Health Cohort Study (CHIWOS), which enrolled WLWH aged ≥16 from British Columbia (BC), Ontario, and Quebec. For this analysis, we excluded women >45 years, who had primary amenorrhea, were pregnant, on hormonal contraception, or who reported history of endometrial cancer, last menstrual period >12 months ago, or premature ovarian failure. The primary outcome was abnormal menstruation (Yes *vs* No) based on responses to five questions about menstrual regularity, frequency, volume, duration, and intermenstrual bleeding in the six months prior to interview. An exploratory multivariable logistic regression analysis examined independent correlates of abnormal menstruation.

**Results:**

Of 1422 women enrolled, 521 (37%) met eligibility criteria. Overall, 55.9% (95% CI:52%-60%) reported abnormal menstruation. In adjusted analyses, abnormal menstruation was associated with having a biologic sister/mother who entered menopause before age 40 (AOR 5.01, 95%CI 1.39–18.03), Hepatitis B co-infection (AOR 6.97, 95%CI 1.52–31.88), current smoking (AOR 1.69, 95%CI 1.55–3.41); and currently taking antiretroviral therapy (ART) (AOR 2.36, 95%CI 1.25–4.45) compared to being ART-naïve. Women in BC had higher adjusted odds of abnormal menstruation (AOR 2.95, 95%CI 1.61–5.39), relative to women in Ontario and Quebec.

**Conclusions:**

Over half of WLWH in this analysis had abnormal menstruation. Correlates of abnormal menstruation include genetic, socio-behavioural factors (province of residence, smoking), Hepatitis B co-infection, and current ART use.

## Introduction

The prevalence of abnormal menstruation is thought to be higher among women living with HIV (WLWH)[1–6], but has not been assessed in nearly two decades. Abnormal menstruation encompasses several variations in the menstrual cycle, including non-menopause related amenorrhea, abnormal cycle frequency, heavy or prolonged menstrual bleeding, and irregular and intermenstrual bleeding[7]. Variations in menstruation are a symptom of multiple conditions, including polyps, adenomyosis, leiomyoma, malignancy and hyperplasia, coagulopathy, ovulatory dysfunction, iatrogenic, endometrial, and not yet classified as potential etiologies causing abnormal menstruation as classified by International Federation of Gynecology and Obstetrics (FIGO) PALM-COIEN system [8].

Abnormal menstruation is thought to be a significant health outcome for WLWH[5,9–14]. Abnormal menstruation is clinically important health outcome, as it is associated with anemia, decreased rates of fertility and reduction in quality of life. Iron-deficiency anemia is a common sequela of heavy menstrual bleeding. The risk of anemia is further compounded with untreated HIV infection, as prevalence of anemia increases with degree of immunosuppression [9]. Increased rates of amenorrhea in WLWH, are proposed to contribute to the decreased rates of fertility found in WLWH [5,10]. WLWH have been found to have an earlier onset of menopause^11^ and disproportionate rates of premature ovarian insufficiency [5,12]. Also, the experience of abnormal menstruation contributes to reduced quality of life for women, with impacts on physical health, psychological wellness and participation in work and social lives [13,14].

The few studies assessing the prevalence of abnormal menstruation in WLWH have shown variable results [1–6]. Early cross-sectional studies found no differences in self-reported rates of amenorrhea in WLWH compared to HIV-negative women [1,2]. A cross-sectional study of Nigerian women with and without HIV observed no significant difference in prevalence of heavy menstrual bleeding or intermenstrual bleeding, however, did observe higher prevalence of amenorrhea and irregular menstrual cycles among WLWH [3]. On the contrary, prospective studies tracking menstrual cycles found that WLWH were more likely to experience menstrual abnormalities, including amenorrhea [4–6] and prolonged menstrual cycle length [5] compared to HIV-negative women.

The mechanisms of abnormal menstruation in WLWH are not clearly understood, however, several risk factors have been identified [11,15,16]. HIV infection and co-morbidities are associated with ovulatory dysfunction, as WLWH who experience abnormal menstruation are found to have decreased levels of follicle stimulating hormone [11,15,16], luteinizing hormone and hyperprolactinemia [16]. Other confounding variables associated with increased rate of abnormal menstruation have included low body mass index (BMI) [2,3,5] and history of a substance use [2,5,17], specifically heroin use among WLWH [5].

There is limited data regarding the association between antiretroviral therapy (ART) use and abnormal menstruation. Massad et al (2006) found low overall rates of abnormal menstruation in WLWH (<20%), yet found that ART use and higher CD4 count was associated with fewer menstrual irregularities [12]. Abnormal menstruation was also found to be more prevalent in WLWH who reported sub-optimal ART adherence and a detectable viral load [18]. Further, it remains unclear whether there is a direct pharmacologic effect of ART on menstrual cycles versus an indirect effect through other pathways.

The purpose of this study was to determine the prevalence of abnormal menstruation in a large cohort of WLWH in Canada. In addition, we assessed correlates of abnormal menstruation with a particular interest on associations with use of ART.

## Methods

### Study overview

This analysis uses data from the baseline survey of the Canadian HIV Women’s Sexual and Reproductive Health Cohort Study (CHIWOS) conducted between 2013 and 2015. CHIWOS is a large community-based research of WLWH aged 16 or older, residing in British Columbia (BC), Ontario, and Quebec. Study design and sampling procedure were published elsewhere [19]. The survey was administered by Peer Research Associates (PRAs), who shared the experience of living with HIV and were hired and trained in community-based research [19]. All participants provided voluntary informed consent at enrollment. CHIWOS was approved by the Research Ethics Boards of Simon Fraser University, University of British Columbia/Providence Health, Women’s College Hospital and McGill University Health Centre.

### Inclusion and exclusion criteria

This analysis was restricted to cisgender women between 16 and 45 years of age who responded to survey questions pertaining to menstruation and ART use, and had at least one period in the last year. We excluded participants who self-reported a history of primary amenorrhea, current pregnancy, regular hormonal contraception use within 6 months of the interview (including oral, transdermal or intramuscular contraceptives, Nuvaring and hormonal intrauterine devices), endometrial cancer, spontaneous menopause (as defined by absence of menstruation for greater than 12 months), or menopause induced by surgery, chemotherapy or radiation.

### Measures

#### Primary outcome

The outcome of interest was abnormal menstruation (Yes vs. No) based on self-report of characteristics of menstrual cycle in the 6 months prior to interview. For the purpose of generalizability to the Canadian population, abnormal menstruation was defined according to the 2013 guidelines of the Society of Obstetricians and Gynecologists of Canada (SOGC, 2013). Although the guidelines were updated in 2018, the 2013 guidelines were in effect at the time of survey completion [7]. Abnormal menstruation was considered present when the participant reported any of five variations from normal menstruation including changes in regularity, frequency, volume, duration and intermenstrual bleeding as defined by the SOGC. Changes in regularity included amenorrhea, defined as “no bleeding in a 90-day period”. Therefore, women who responded that most recent menstrual period occurred “more than 3 months ago” were defined as having abnormal menstruation, with amenorrhea. Changes in frequency were defined as abnormal if menstrual cycles were (1) frequent with “bleeding at intervals <24 days apart” or (2) infrequent with “bleeding at intervals >38 days apart” or (3) irregular frequency with “a range of varying lengths of bleeding free intervals exceeding 20 days.” Therefore, women who responded that length of cycles were “less than 24 days” or “greater than 35 days” or “too variable or irregular to say” were classified as having abnormal menstruation. Changes in volume with heavy menstrual bleeding were defined as “excessive menstrual blood loss which interferes with the woman’s physical, emotional, social and material quality of life, and which can occur alone or in combination with other symptoms.” Therefore, women who reported experiencing heavy menses by responding that in the last 6 months their menstrual bleeding has been “heavy or very heavy” were classified as experiencing abnormal menstruation with heavy menstrual bleeding. Variation in duration with “menstrual blood loss which exceeds 8 days in duration” was classified as prolonged menstrual bleeding. Therefore, women who responded that the duration of menstrual period as “greater than 7 days” in the last six months were defined as experiencing abnormal menstruation with prolonged menstrual bleeding. Intermenstrual bleeding was defined as “irregular episodes of bleeding, often light and short, occurring between otherwise fairly normal menstrual periods.” Women who responded that they had spotting or bleeding between menstrual periods were classified as having irregular menses. The specific questions regarding menstruation and menstrual abnormalities are included in **S1 Appendix:** The primary outcome was a composite outcome of abnormal menstruation, such that if the participant reported ***at least one*** abnormal characteristic of her menstrual cycle (as described above), then she was classified as having abnormal menstruation.

#### Correlates

Socio-demographic, socio-behavioral, and clinical variables, including co-infections and ART use, were correlates of interest to abnormal menstruation in this analysis. Socio-demographic characteristics including age, BMI, ethnicity, birth country, and province of interview (i.e., British Columbia, Ontario or Quebec) were considered. Ethnicity was grouped as Indigenous, African/Caribbean/Black, white, or Other ethnicities. Biological first degree relative (sister or mother) who entered menopause before age 40, was included to capture underlying genetic predisposition to early menopause. Socio-behavioural variables used in this analysis were annual household income (<$20,000 vs ≥$20,000), education (< high school education or high school education or greater), and food security. Food security was deemed insufficient if participants responded, “Sometimes true” or “Often true” when asked if they (i) worried food would run out before you got money to buy more OR (ii) food bought did not last and there was not any money to get more OR (iii) could not afford to eat balanced meals. Conversely, food security was deemed sufficient if participants responded, “Never true” to the three aforementioned questions. Clinical variables included were duration of HIV diagnosis, self-reported most recent CD4 count and viral load, injection drug use, cigarette use, previous or current hepatitis B and C co-infection, and ART history. Substance use and cigarette were grouped as current, previous, never use. We assessed self-reported use of ART including: (1) ART use (current use of any class of ART vs. previous but not current ART use vs ART-naive) (2) self-reported current class of nucleoside reverse transcriptase inhibitor (NRTI) used, and (3) self-reported current class of 3^rd^ agent ART used.

### Statistical analyses

Descriptive statistics, with medians and interquartile ranges (IQRs) for continuous variables and frequencies and proportions for categorical variables, were used to describe the socio-demographic, socio-behavioural, and clinical characteristics of the analytic sample and report the prevalence of abnormal menstruation overall and the prevalence of each symptom (e.g. amenorrhea, menorrhagia). Participants with and without abnormal menstruation were compared using Chi-square or Fisher’s Exact test for categorical variables and Wilcoxon rank-sum test for continuous variables. Correlates which were statistically significantly (p-value <0.05) associated with abnormal menstruation in the bivariable analyses were considered for the multivariable logistic regression analysis. The final model was selected based on a modified step-wise backward selection process balancing between significance of covariates (Type III p-value) and the model goodness-of-fit [20].

## Results

### Study sample

Of the 1422 total CHIWOS baseline participants, 521 were eligible for this analysis (37% of total cohort). Reasons for exclusion were age >45 (n = 571), never had menstrual period (i.e. primary amenorrhea) (n = 55), past history of hysterectomy or oophorectomy (n = 45), past history of chemotherapy or radiation (n = 13), current pregnancy (n = 20), hormonal contraception use in the last 6 months (n = 133) or history of endometrial cancer (n = 2). Participants were also excluded if they did not respond to questions regarding menstruation (n = 35) or if they identified as post-menopausal (n = 27) (defined via self-report of experiencing spontaneous or induced menopause or having had her last menstrual period over 12 months ago).

### Prevalence of abnormal menstruation

The prevalence of abnormal menstruation was 55.9% (95% confidence interval (CI), 51.6% - 60.1%) (n = 291). Six percent (95%CI 3.9%-8.0%) (n = 31) of the participants were amenorrheic (i.e. did not have a menstrual period in the last 3 months). Abnormal cycle length was reported by 33.8% of women (95%CI 30.8%-39.0%). 31.0% reported increased menstrual volume with heavy or very heavy menstrual bleeding. Other menstrual symptoms characterized in the cohort include irregular/prolonged menstruation (8.3%) (95%CI 7.3%-9.8%) and intermenstrual bleeding (13.6%) (95%CI 15.4%-22.2%). The menstruation characteristics of the study cohort are shown in **Table 1**.

**Table 1:** Prevalence of normal vs. abnormal menstruation characteristics in the last 6 months for women in CHIWOS cohort (N = 521).

		*Total* *N = 521* *N (%)*	
Overall menstruation history in the past 6 months*			
Normal menstruation		230	(44.1)
Abnormal menstruation		291	(55.9)
**Individual measures of menstruation history in the past 6 months**			
**Regularity: Amenorrhea**			
Normal (most recent menstrual period within 3 months)		490	(94.0)
Abnormal (most recent menstrual period more than 3 months ago)		31	(6.1)
**Frequency/Regularity: Menstrual cycle length**			
	Normal (24–35 days)	329	(63.1)
	Abnormal (<24 days, >35 days, too irregular to say)	173	(33.8)
	DK/PNTA length	10	(1.9)
**Volume: Heavy Menstrual Bleeding**			
	NORMAL Light	40	(7.7)
	Medium	287	(55.1)
	ABNORMAL	165	(31.0)
	Heavy	86	(16.5)
	Very heavy	43	(8.3)
	Too irregular to say	36	(6.9)
	DK/PNTA	2	(0.4)
**Duration: Prolonged menstrual bleeding**			
	NORMAL Less than 4 days	107	(19.7)
	Between 4–7 days	339	(62.5)
	ABNORMAL Greater than 7 days	21	(3.9)
	Too irregular to say	24	(4.4)
	DK/PNTA	3	(0.6)
**Irregular: Intermenstrual bleeding**			
	Normal (No)	420	(80.6)
	Abnormal (Yes)	71	(13.6)
	DK/PNTA	3	(0.6)

DK/PNTA, Don’t Know or Prefer Not to Answer

Note:

*If participant reported ***at least one*** abnormal characteristic of her menstrual cycle as shown below, then she was classified as having ‘Abnormal Menstruation’. Otherwise, she is classified as Normal Menstruation.

### Bivariable correlations with abnormal menstruation

Socio-demographic, socio-behavioural and clinical characteristics of the included participants are shown in **Table 2**. The median age was 37 years (IQR 33.0, 41.0), and the median time since HIV diagnosis was 8.6 years (IQR 4.4, 13.4 years). The majority of women (79.3%) were currently taking ART, while 4.8% previously used ART, and 15.5% never used ART. The most commonly reported nucleoside reverse transcriptase inhibitors (NRTI) used were Truvada^®^ (emtricitabine/tenofovir) (42.0%) and Kivexa^®^ (abacavir/lamivudine) (19.0%). Protease inhibitors (PIs) (32.2%) and non-nucleoside reverse transcriptase inhibitors (NNRTIs) (24.2%) were the most common third agent classes used. Only 33 women (6.3%) reported taking an integrase inhibitor. In total, 72.7% (n = 379) of women reported an undetectable viral load (<50 copies/mL); 93% in those on ART.

**Table 2:** Prevalence of correlates in normal vs. abnormal menstruation in the last 6 months for Canadian women living with HIV in CHIWOS cohort (N = 521).

*Characteristic*		*Overall* *N = 521* *N(%)or* *Median [IQR]*	*Normal* *N = 230* *N(%)or* *Median [IQR]*	*Abnormal* *N = 291* *N (%) or Median [IQR]*	*P–value*
**Age**					
	20 or less	8 (1.5)	2 (25.0)	6 (75.0)	**0.001**
	21 to 29	66 (12.7)	34 (51.5)	32 (48.5)	
	30 to 39	272 (52.2)	137 (50.4)	135 (49.6)	
	40 or more	175 (33.6)	57 (32.6)	118 (67.4)	
	Median Age	37.0 [33.0–41.0]	36 [IQR 32.0–39.0]	38 [IQR 33.0–42.0]	**0.003**
**BMI**					
	Underweight (<18.5)	25 (4.8)	10 (40.0)	15 (60.0)	0.704
	Normal (18.5–25)	221 (42.4)	98 (44.3)	123 (55.7)	
	Overweight (25–30)	134 (25.7)	61 (45.5)	73 (54.5)	
	Obese (>30)	118 (22.6)	50 (42.4)	68 (57.6)	
**Ethnicity**					
	Indigenous	114 (21.9)	48 (42.1)	66 (57.9)	0.859
	African/ Caribbean/ Black	200 (38.4)	88 (44.0)	112 (56.0)	
	White	170 (32.6)	79 (46.5)	91 (53.5)	
	Other ethnicities	37 (7.1)	15 (40.5)	22 (59.5)	
**Biological Sister/Mother Who Entered Menopause before 40**					
	No	394 (75.6)	200 (50.8)	194 (49.2)	**<0.001**
	Yes	26 (5.0)	3 (11.5)	23 (88.5)	
	DK/PNTA*	101 (19.3)	27 (26.7)	74 (73.3)	
**Province that Interview was Conducted**					
	British Columbia	117 (22.5)	26 (22.2)	91 (77.8)	**<0.001**
	Ontario	287 (55.0)	161 (56.1)	126 (43.9)	
	Quebec	117 (22.5)	43 (36.8)	74 (63.2)	
**Household Income**					
	< $20 000	317 (60.8)	126 (39.7)	191 (60.3)	**0.019**
	$20 000 or more	184 (35.3)	93 (50.5)	91 (49.5)	
	DK/PNTA*	20 (3.8)	11 (55.0)	9 (45.0)	
**Education**					
	< High school	70 (13.4)	19 (27.1)	51 (72.9)	**0.021**
	High school or greater	449 (86.2)	211 (47.0)	238 (53.0)	
**Food Security**					
	Sufficient	67 (29.0)	79 (52.8)	60 (47.2)	**0.028**
	Insufficient	163 (52.0)	119 (41.6)	229 (58.4)	
**Duration of HIV Diagnosis**					
	Less than 5 years	142 (27.3)	78 (54.9)	64 (45.1)	**<0.001**
	5–10 years	144 (27.6)	67 (46.5)	77 (53.5)	
	Greater than 10 years	215 (41.3)	73 (34.0)	142 (66.0)	
	DK/PNTA*	20 (3.8)	12 (60.0)	8 (40.0)	
	Median Age				
**Most Recent CD4**					
	< 350	56 (10.7)	19 (33.9)	37 (66.1)	0.151
	> = 350	305 (58.5)	135 (44.3)	170 (55.7)	
	DK/No CD4 Result/PNTA*	160 (30.7)	76 (47.5)	84 (52.5)	
**Most Recent Viral Load**					
	Undetectable (< 50)	379 (72.7)	157 (41.4)	222 (76.3)	0.147
	Detectable (> = 50)	86 (16.5)	43 (50.0)	43 (14.8)	
	DK/No VL Result/PNTA*	56 (10.7)	30 ()	26 (9.0)	
**Cigarette Use**					
	Current Smoker	215 (41.2)	67 (31.2)	148 (68.8)	**<0.001**
	Previous Smoker	39 (7.4)	20 (51.3)	19 (48.7)	
	Never Smoker	262 (50.3)	142 (54.2)	120 (45.8)	
	DK/PNTA*	5 (1.0)	1 (20.0)	4 (80.0)	
**Injection Drug Use**					
	Current Injection Drug User	48 (9.2)	10 (20.8)	38 (79.2)	**<0.001**
	Previous Injection Drug User	89 (17.1)	23 (25.8)	66 (74.2)	
	Never Injection Drug User	377 (72.3)	193 (51.2)	184 (48.8)	
	DK/PNTA*	7 (1.3)	4 (57.1)	3 (42.9)	
**Hepatitis B Co-infection (Previous or Active)**					
	No	23 (4.4)	226 (45.9)	266 (54.1)	**0.002**
	Yes	492 (94.4)	3 (13.0)	20 (87.0)	
	DK/PNTA*	6 (1.2)	1 (16.7)	5 (83.3)	
**Hepatitis C Co-infection (Previous or Active)**					
	No	138 (26.5)	188 (49.6)	191 (50.4)	**<0.001**
	Yes	379 (72.7)	40 (29.0)	98 (71.0)	
	DK/ PNTA*	4 (0.8)	2 (50.0)	2 (50.0)	
**ART Use**					
	Never on ART	81 (15.5)	55 (67.9)	26 (32.1)	**<0.001**
	Previously on ART	25 (4.8)	10 (40.0)	15 (60.0)	
	Currently on ART	413 (79.3)	163 (39.5)	250 (60.5)	
	DK/PNTA *	2 (0.4)	2 (100.0)	0 (0)	
**Current NRTI Used**					
	Truvada	219 (42.0)	89 (40.6)	130 (59.4)	0.886
	Kivexa	99 (19.0)	34 (34.3)	65 (65.7)	
	Combivir/Trizivir/Other NRTI Backbone	34 (6.6)	15 (44.1)	19 (55.9)	
	No NRTI Backbone	61 (11.7)	25 (41.0)	36 (59.0)	
	Not currently on ART	106 (20.3)	65 (61.3)	41 (38.7)	
	DK/PNTA*	2 (0.4)	2 (100)	0 (0)	
**Current Class of 3**^**rd**^ **Agent ART**					
	PI	168 (32.2)	61 (30.6)	107 (63.7)	0.560
	NNRTI	126 (24.2)	57 (45.2)	69 (54.8)	
	Integrase Inhibitor	33 (6.3)	13 (39.4)	20 (60.6)	
	Other Regimens	68 (13.0)	32 (41.0)	36 (59.0)	
	No 3^rd^ Agent	25 (4.8)	7 (3.0)	18 (6.2)	
	Not currently on ART	106 (20.3)	65 (61.3)	41 (38.7)	
	DK/PNTA*	2 (0.4)	2 (100.0)	0(0)	

*DK/PNTA not included in the p-value calculations.

BMI, Body mass index; DK/PNTA, Don’t Know or Prefer Not to Answer; VL, Viral load; ART, antiretroviral therapy, NRTI, nucleoside Reverse Transcriptase Inhibitor; PI, protease inhibitor; NNTRI, non-nucleoside reverse transcriptase inhibitors

Age, duration of HIV diagnosis, ART use, current CD4 count, injection drug use, cigarette use, hepatitis B and C co-infection, province of residence, education, food security and income were associated with abnormal menstruation in bivariate analyses (**Table 2**). Abnormal menstruation was associated with older age with a median age of 38 (IQR 33.0–42.0, p = 0.003), compared to a median age of 36 (IQR 32.0–39.0) in women who did not report abnormal menstruation. Women with abnormal menstruation also had an increased duration of HIV diagnosis with a median duration of 10.2 years (IQR 5.5–14.2, p = 0.001). Current ART use as compared to never being on ART was found to correlate with increased rates of abnormal menstruation (60.5% vs. 32.1%, *p*<0.001). Of the classes of antiretroviral drugs used, no significant difference was found between the different current NRTI used or various current 3^rd^ agent class. Increased rates of abnormal menstruation correlated with current injection drug use as compared to those who have never used injection drugs (79.2% vs. 48.8%, *p*<0.001). Increased rates of abnormal menstruation were observed in women with hepatitis B (87.0% vs. 54.1%, p = 0.002) and hepatitis C (71.0% vs. 50.4%, p<0.001) co-infection as compared to women without hepatitis B or C.

The prevalence of abnormal menstruation among women from BC was 78.0%, compared to 44.0% in Ontario and 63.0% in Quebec (p-value <0.001). Abnormal menstruation was significantly higher among women with lower education (72.9%) compared to 53% among women with a high school education or higher, food insecurity (58.4%) compared with 47.2% among women with sufficient food security, household income <$20,000 per year (60.3%) compared with 49.5% among women with a household income > = $20,000 per year. Women who had a biologic sister or mother who entered menopause before 40 had a higher significantly prevalence of abnormal menstruation than those who did not (88.5% vs. 49.2%).

### Bivariable and multivariable logistic regression

Bivariable and multivariable logistic regression analyses are presented in **Table 3**. The final set of variables included in the multivariable model were age, duration of HIV diagnosis, biological first degree relative who entered menopause before age 40, province of interview, ART use, hepatitis B infection, and cigarette smoking. In the adjusted model, factors associated with increased odds of abnormal menstruation included: having a biologic sister or mother who entered menopause before 40 (AOR 5.00, 95%CI 1.39–18.03), current smoking (AOR 2.30, 95%CI: 1.55–3.41 compared to women who have never smoked), current ART use (AOR 2.36 95%CI: 1.25–4.45 compared to ART-naïve), current or previous hepatitis B co-infection (AOR 6.97 95%CI 1.52–31.88), and province of interview (BC vs Ontario AOR 2.95, 95%CI: 1.61–5.39; Quebec vs Ontario AOR 1.90, 95%CI: 1.13–3.09). Although not significant at p<0.05, women with a household income of less than $20 000 (AOR 1.52, 95%CI 0.98–2.37) had greater adjusted odds of abnormal menstruation compared to women with income levels $20 000 or more. There was no significant association between time since HIV diagnosis and adjusted odds of abnormal menstruation.

**Table 3:** Logistic regression analyses for clinical and sociodemographic correlates with normal and abnormal menstruation in the last 6 months in Canadian women living with HIV in CHIWOS cohort (n = 521).

Variable	Unadjusted		Adjusted	
	OR (95%CI)	P-value	OR (95%CI)	P-value
**Age at Interview Date**	1.04 (1.01, 1.08)	**0.006**	1.03 (1.00, 1.07)	**0.07**
**Duration of HIV Infection (months)**	1.004 (1.001, 1.007)	**0.003**	1.002 (0.999, 1.005)	**0.147**
**Biological Mother/Sister who entered natural menopause before age 40**		**<0.001**		**0.001**
No	1.00 (-)		1.00 (-)	**<0.001**
Yes	7.46 (2.19, 25.37)		5.01 (1.39, 18.03)	
Don’t Know	3.05 (1.80, 5.16)		2.53 (1.44, 4.45)	
**Most Recent CD4**		**0.232**	Not Selected	
<350	1.00 (-)			
> = 350	0.65 (0.35, 1.21)			
Does not know	0.56 (0.28, 1.09)			
**Cigarette use**		**<0.001**		**0.017**
Never	1.00 (-)		1.00 (-)	
Former	0.96 (0.47, 1.93)		0.61 (0.27, 1.40)	
Current	2.30 (1.55, 3.41)		1.69 (1.06, 2.68)	
**Injection drug use**		**<0.001**	Not Selected	
Never	1.00 (-)			
Previous use	2.88 (1.70, 4.90)			
Current use	4.53 (1.94, 10.57)			
**Hepatitis B**		**0.008**		**0.012**
No	1.00 (-)		1.00 (-)	
Yes	7.38 (1.69, 32.20)		6.97 (1.52, 31.88)	
**Hepatitis C**		**<0.001**	Not Selected	
No	1.00 (-)			
Yes	2.55 (1.62, 4.01)			
**ART use**		**<0.001**		**0.030**
Never on ARTs	1.00 (-)		1.00 (-)	
Previous ART	3.90 (1.39, 10.95)		2.11 (0.67, 6.43)	
Currently on ART	4.20 (2.41, 7.33)		2.36 (1.25, 4.45)	
**Province Interview Conducted**		**<0.001**		**0.001**
Ontario	1.00 (-)		1.00 (-)	
British Columbia	4.83 (2.84, 8.21)		2.95 (1.61, 5.39)	
Quebec	2.26 (1.42, 3.60)	** **	1.9 (1.13, 3.09)	
**Household gross yearly income**		**0.040**		**0.061**
$20 000 or more	1.00 (-)		1.00 (-)	
< $20 000	1.49 (1.02, 2.17)		1.52 (0.98, 2.37)	
**Education**		**0.003**	Not Selected	
Less than high school	1.00 (-)			
High school or greater	0.38 (0.20, 0.71)			
**Food Security**		**0.151**	Not Selected	
Sufficient	1.00 (-)			
Insufficient	1.37 (0.89, 2.11)			

ART, antiretroviral therapy, VL, viral load

## Discussion

Over half (56%) of WLWH in our study reported abnormal menstruation. In previous studies, there have been variable results in the association between HIV and abnormal menstruation. Ezechi et al., found increased rates of amenorrhea and irregular menstrual cycles in WLWH compared to HIV-negative controls in a Nigerian population [3]. Initial prospective trials also found increased rates of amenorrhea [4–6], short menstrual cycles [6], decreased cycle frequency [5] as well as early onset menopause [11]. Massad et al., compared the prevalence of amenorrhea, skipped periods, intermenstrual bleeding, and menorrhagia in WLWH to HIV negative controls, and found low prevalence of abnormal menstruation (less than 20%) [12]. However, most of the research in menstrual abnormalities occurred in the mid-1990s and early 2000s, where management of HIV greatly differed and patients had more advanced disease. In our study, correlates of abnormal menstruation included current a first degree relative who had premature menopause, ART use, cigarette use, hepatitis B co-infection and province that interview was conducted.

The mechanisms underlying possible abnormal menstruation in WLWH are yet to be clearly delineated. Many confounding variables can account for secondary amenorrhea in adult women. Of these, age, injection drug use, and biological mother or sister who entered menopause before 40 years were accounted for in our model. Increasing age and concurrent injection drug use has been demonstrated to contribute to abnormal menstruation [2,5,11,18], and showed significant associations in unadjusted models but were not selected for the multivariable model. In this study, we characterized ART use, cigarette use, hepatitis B co-infection and province that interview was conducted as the significant correlated for abnormal menstruation in women living with HIV.

The symptoms contributing to abnormal menstruation were primarily abnormal cycle length (33.8%), amenorrhea (6.1%), intermenstrual bleeding (13.6%), and heavy menstrual bleeds (31.0%). The prevalence of secondary amenorrhea (6.1%) is a clinically important finding from our analyses. Amenorrhea in pre-menopausal women can reduce fertility [5,10], which can be distressing to young women who have not yet completed their families. Previous studies have also demonstrated increased rates of amenorrhea in WLWH compared to women without HIV [3–5,18]. These changes to menstrual cycles in WLWH may be associated with early onset of menopause [11]. Early onset of menopause is associated with increased risk of cardiovascular disease, diabetes, and osteoporosis [21]. Our study also found significant rates of heavy menstrual bleeding, which is clinically important in the context of HIV results in a compounded risk of developing anemia. Anemia in itself can cause a reduction in functional capacity and quality of life.

Interestingly, province of interview was associated with abnormal menstruation. Participants in BC reported higher rates of menstrual abnormality as compared to Ontario and Quebec. The provincial differences in prevalence of abnormal menstruation are likely multifactorial. In part, variation in province are secondary to socio-economic factors, including composite measure of life instability, including socio-economic status, injection drug use and ethnicity that were not captured with a single variable. Moreover, our analysis did not account for variation in healthcare resources by province. Healthcare delivery varies between provinces and within health regions, including access to primary care physicians and specialists. As such, regions that have higher access to specialized medical centres may have more timely and comprehensive investigations regarding abnormal menstruation. Further, specialist care may be required for definitive treatment of underlying causes of abnormal menstruation, such as fibroid surgery. Lastly, pharmaceutical coverage of medications may vary between provinces as well. As such, province of interview, encompasses multiple variables that determine access to healthcare.

Consistent with others, we found that women reporting current cigarette use had 70% higher odds of abnormal menstruation compared to women who have never smoked. Cigarette use has been demonstrated to be associated with abnormal menstruation, including heavy, prolonged, irregular and intermenstrual bleeding [22]. Moreover, a cigarette dose response has been demonstrated, with the highest rates of irregular menstruation and heavy periods observed in women who smoked 20 or more cigarettes per day among women without specified HIV status [23]. Cigarette smoking is associated with hypoestrogenism [24]. It is proposed that nicotine interferes with steroid synthesis and promotes androgen excess [25], causing anovulation and menstrual abnormalities, including breakthrough bleeding and spotting [26]. Further, it is proposed that cigarette smoking may promote abnormal angiogenesis increasing the risk of abnormal bleeding [27,28].

The association between self-reported hepatitis B with abnormal menstruation has not previously been demonstrated. A proposed mechanism is that hepatitis B co-infection resulting in liver disease and associated platelet dysfunction would cause heavy or prolonged menstrual bleeding. As found in other studies, injection drug use was associated with an increase in abnormal menstruation [5,11,18]. Chirgwin (1996) and Harlow (2003) found an association with increased rates of amenorrhea with heroine and/or methadone use [5,18]. Opioid use in particular is known to suppress the hypothalamic-pituitary access, which may explain the high rate of amenorrhea in our cohort. Moreover, it is noteworthy that women who reported previous, but not current injection drug use also experience higher rates of abnormal menstruation, which suggests that injection drug use may cause long term disruption of menstrual cycles. Interestingly, low BMI has been previously demonstrated to disrupt pituitary axis result in amenorrhea and has been demonstrated in WLWH [2,3], but has not been shown to be a significant correlate to abnormal menstruation in our analysis. However, only a 4.8% of the cohort were classified as having a low BMI (<18.5), but within this subgroup there was a positive association with low BMI and abnormal menstruation.

Current ART use was found to correlate with increased odds of abnormal menstruation compared with ART-naïve women. In a previous study, Massad et al. concluded that ART use was correlated with lower incidence of menstrual irregularity [12]. However, these conclusions may be unreliable, as only 16% of the HIV-positive women in this study had ever initiated ART [12]. Also, Massad et al. found low overall rates of abnormal menstruation (<20%) in an American population of women living with HIV [12], which drastically differs from the rate of 56% in the CHIWOS cohort. Although this study by Massad et al., indicated an association between ART use and improved menstrual regularity and flow [12], the low rate of ART use in the population may yield invalid results and may not reflect the current population of WLWH. Further, we observed no independent correlation between abnormal menstruation and CD4 count or years since HIV diagnosis suggesting that the effect of ART on menstrual irregularity is likely a drug effect rather than sequelae of HIV infection. Of the specific self-reported antiretroviral drugs used, bivariate analyses demonstrated no difference between the use of NRTI or class of 3^rd^ agent class used. Further, participants are asked to report their current treatment regimen, therefore we cannot account for crossover effect of prior treatments. Another challenge is that there is no information on dosing or duration of ART treatment.

Limitations in our study include that our survey used self-report of menstrual cycle experiences and all other variables. For example, menorrhagia was defined according to experience of ‘heavy menstrual bleeding’ rather quantifying blood loss[1]. However, the SOGC defines heavy menstrual bleeding as “excessive menstrual blood loss which interferes with the woman’s physical, emotional, social and material quality of life, and which can occur alone or in combination with other symptoms [7].” Therefore, the experience of “heavy or very heavy” menstruation is clinically useful for characterizing patients’ symptoms. A limitation is the exclusion criteria for our analysis. First, we did not explicitly exclude post-partum or breastfeeding women in our study, which may lead to overestimating the prevalence of amenorrhea or intermenstrual bleeding. However, we excluded women who were currently pregnant and women who did not have their period in the last year, which should exclude most women who are post-partum. Further, we did not collect data on infant feeding practices, however, given Canadian guidelines recommending against breastfeeding for women living with HIV, we expect the proportion of participants reporting breastfeeding to be very low. Also, we excluded women who were using hormonal contraception or IUD, which may exclude participants who used these products for regulation of abnormal menstruation. In our analysis of contraceptive use among women living with HIV in the CHIWOS study [29], we asked women who reported using the oral contraceptive pill, injectable hormonal contraception, and the contraceptive implant the reasons for using these methods. Women could report more than one reason. Overall, over half of women using the oral contraceptive pill and a third of women using injectables reported using these methods to “regulate menstrual periods and/or menstrual symptoms” only or in addition to contraceptive effects. By excluding women on hormonal contraception from our analysis, we may have under-estimated the proportion of women living with HIV experiencing abnormal menstruation. Moreover, the co-variates were obtained through participant self-reported data, and therefore there is a risk of recall bias for variables such as CD4 count, viral load and time since HIV infection (which may be longer than time since HIV diagnosis). Further, we investigated abnormal menstruation as a composite of multiple symptoms, such as amenorrhea and heavy menstrual bleeding. We could not characterize the etiology of these symptoms, as we did not differentiate if the cause of pathology was attributed to fibroids, malignancy, coagulopathy or endocrine changes.

In conclusion, we found that over half of all WLWH in our study reported abnormal menstruation. Abnormal menstruation is associated with co-morbidities such as anemia and affects women’s quality of life. Also, increased rates of menstrual abnormalities may contribute to decreased rates of fertility. The fact that we found high rates of abnormal menstruation among a large population of WLWH is significant to the community and clinicians. Clinicians should be prompted to offer hormonal contraceptive methods including intrauterine devices for management of anemia, quality of life and cycle regulation if reporting symptoms of heavy or prolonged menstruation. Abnormal menstruation and the impact on fertility and family planning are to be further investigated. Additionally, clinicians should be aware of risks of early menopause and associated co-morbidities in WLWH.

## Supporting information

S1 Appendix Definition of abnormal menstruation and normal menstruation based on Society of Obstetricians and Gynecologists of Canada (2013) definitions of abnormal menstruation and corresponding participant responses on CHIWOS survey. (DOCX)
