# Supplementary material for: High prevalence of abnormal menstruation among women living with HIV in Canada
Source: PLoS One. 2019 Dec 27;14(12):e0226992. doi: 10.1371/journal.pone.0226992 (PMC6934328; doi:10.1371/journal.pone.0226992)
Supplement: S1 Appendix — (DOCX) [file pone.0226992.s001.docx]

| **Appendix 1: Definition of Abnormal Menstruation** | | |
| --- | --- | --- |
| **Menstrual cycle characteristics in the last 6 months and related survey questions** | **CHIWOS Survey Question** | **Definition of Normal Menstruation**  **Vs. Abnormal Menstruation** |
| **Regularity: Amenorrhea**  **Clinical Definition of Variable:** No bleeding in a 90-day period [SOGC 2013] | When did you start your most recent menstrual period? | **Normal Menstruation**   - Within the last month - More than 1 month ago, but within the last 3 months   **Abnormal Menstruation**   - More than 3 months ago   **Missing:**   - DK/PNTA |
| **Frequency/Regularity: Menstrual cycle length**  **Clinical Definition of Variable:**  Infrequent: Bleeding at intervals >38 days apart. Frequent Bleeding at intervals <24 days apart. Irregular bleeding: A range of varying lengths of bleeding free intervals exceeding 20 days [SOGC 2013] | In the last six months, what was the usual length of your menstrual cycles? | **Normal Menstruation**   - Between 24-35 days   **Abnormal Menstruation**   - Infrequent: Less than 24 days - Frequent: Greater than 35 days - Irregular: Too variable or irregular to say   **Missing:**   - DK/PNTA length |
| **Volume: Heavy Menstrual Bleeding**  **Clinical Definition of Variable:**  Excessive menstrual blood loss which interferes with the woman’s physical, emotional, social and material quality of life, and which can occur alone or in combination with other symptoms.  [SOGC 2013] | How would you describe your menstrual flow in the last six months?  My menstrual bleeding has been or was: | **Normal Menstruation**   - Light OR Medium   **Abnormal Menstruation**   - Heavy OR Very Heavy - Too irregular to say   **Missing:**   - DK/PNTA |
| **Duration: Prolonged Menstrual Bleeding**  **Clinical Definition of Variable:** Describes menstrual blood loss which exceeds 8 days in duration [SOGC 2013] | Which of the following describes the duration of your menstrual period in the last six months? | **Normal Menstruation**   - Less than 4 days - Between 4-7 days   **Abnormal Menstruation**   - Greater than 7 days - Too irregular to say   **Missing:**   - DK/PNTA |
| **Irregular, non-menstrual: Intermenstrual Bleeding**  **Clinical Definition of Variable:**  Irregular episodes of bleeding, often light and short, occurring between otherwise fairly normal menstrual periods. [SOGC 2013] | In the last six months, have you had spotting or bleeding between menstrual periods? | **Normal Menstruation**   - No   **Abnormal Menstruation**   - Yes   **Missing:**   - DK/PNTA |
| **Primary Outcome: Overall menstruation history in the last 6 months** | **Normal Menstruation**   - If participant reported normal menstruation in all of the characteristics of her menstrual cycle, then she is classified as having “Normal Menstruation”   **Abnormal Menstruation**   - If participant reported ***at least one*** abnormal characteristic of her menstrual cycle, then she is classified as having Abnormal Menstruation | |
